# Biomechanical testing of fixed and adjustable femoral cortical suspension devices for ACL reconstruction under high loads and extended cyclic loading

**DOI:** 10.1186/s40634-020-00235-9

**Published:** 2020-05-11

**Authors:** Sarvpreet Singh, Sonia Ramos-Pascual, Kinga Czerbak, Muzaffar Malik, Peter J. Schranz, Anthony W. Miles, Vipul Mandalia

**Affiliations:** 1 Exeter Knee Reconstruction Unit, Princess Elizabeth Orthopaedic Centre, Royal Devon and Exeter Hospital, Exeter, UK; 2 Centre for Orthopaedic Biomechanics, Department of Mechanical Engineering, University of Bath, Bath, UK; 3 Division of Medical Education, Brighton and Sussex Medical School, University of Brighton, BN1 9PH Brighton, UK

**Keywords:** Anterior cruciate ligament reconstruction, Fixed-loop femoral cortical suspension device, Adjustable-loop femoral cortical suspension device, Biomechanical testing, High loads, Extended cyclic loading

## Abstract

**Purpose:**

To compare loop elongation after 5000 cycles, loop-elongation at failure, and load at failure of the fixed-loop G-Lok device and three adjustable-loop devices (UltraButton, RigidLoop Adjustable and ProCinch RT), during testing over extended cycles under high loading.

**Methods:**

Five devices of each type were tested on a custom-built rig fixed to an Instron machine. The testing protocol had four stages: preloading, cyclic preconditioning, incremental cyclic loading and pull-to-failure. Outcome measures were loop elongation after 5000 cycles, loop-elongation at failure, and load at failure.

**Results:**

The loop elongation after 5000 cycles for G-Lok was 1.46 ± 0.25 mm, which was comparable to that of RigidLoop (1.51 ± 0.16 mm, *p* = 1.000) and ProCinch (1.60 ± 0.09 mm, *p* = 1.000). In comparison, the loop elongation for UltraButton was 2.66 ± 0.28 mm, which was significantly larger than all other devices (*p* = 0.048). The failure load for all devices ranged between 1455 and 2178 N. G-Lok was significantly stronger than all adjustable-loop devices (*p* = 0.048). The elongation at failure was largest for UltraButton (4.20 ± 0.33 mm), which was significantly greater than G-Lok (3.17 ± 0.33 mm, *p* = 0.048), RigidLoop (2.88 ± 0.20 mm, *p* = 0.048) and ProCinch (2.78 ± 0.08 mm, *p* = 0.048). There was no significant difference in elongation at failure for the rest of the devices.

**Conclusions:**

Our study has shown that the G-Lok fixed-loop device and the three adjustable-loop devices (UltraButton, RigidLoop Adjustable and ProCinch RT) all elongated less than 3 mm during testing over an extended number of cycles at high loads, nonetheless, the fixed loop device performed best in terms of least elongation and highest load at failure.

## Background

Symptomatic knee instability after anterior cruciate ligament (ACL) injury may require reconstruction of the ACL with an auto- or allo- graft, which is fixed to the tibia and femur using interference screws, transfixation pins or cortical suspension loop devices [15, 20, 22, 36]. The most suitable femoral fixation technique is debatable, but cortical suspension fixed-loop devices give good, reproducible results [1].

The more recent cortical suspension adjustable-loop devices have several advantages: (1) they are easier to use in short femoral tunnels, with placement through the antero-medial arthroscopic portal; (2) they allow more of the femoral tunnel to be filled with graft, and shorter graft lengths can be used, as seen with tripling/quadrupling of the graft; (3) they are suitable for most tunnel sizes, eliminating the need for fixed-loop devices with different loop sizes [9, 14, 16, 18]. However, there are concerns about the elongation of cortical suspension adjustable-loop devices under cyclic loading post-fixation, which relate to the button-locking mechanism [26]. Studies show that cortical suspension fixed-loop devices elongate less than adjustable-loop devices, the latter has been shown to elongate by more than 3 mm, which introduces knee instability and is regarded as a clinical failure [13, 19].

There have been a variety of studies investigating the elongation and failure load of fixed-loop cortical suspension devices in vitro, however, to the authors’ knowledge there has been only one study testing the fixed-loop G-Lok device, and this was performed under low loads and a low number of cycles. The aim of this study was to compare the loop elongation after 5000 cycles, the loop-elongation at failure, and the load at failure of the fixed-loop G-Lok device and three adjustable-loop devices (UltraButton, RigidLoop Adjustable and ProCinch RT), during testing over an extended number of cycles under high loading. The authors hypothesised that the fixed-loop device would have a lower elongation after 5000 cycles and at failure, as well as a higher failure load, than the adjustable-loop devices.

## Methods

**Fig. 1:**
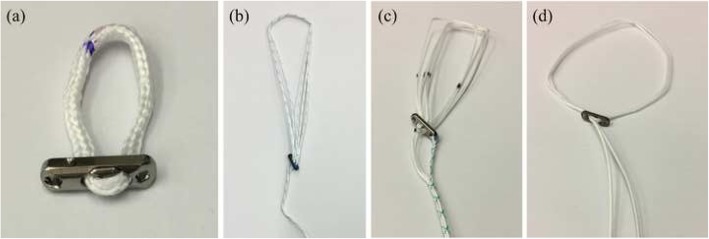
Fixed and adjustable femoral cortical suspension devices tested in the current study: **a** G-Lok (Stryker Sports Medicine, Greenwood Village, Colorado, USA), **b** UltraButton (Smith & Nephew, Andover, Massachusetts, USA), **c** RigidLoop Adjustable (DePuy Mitek, Raynham, Massachusetts, USA), and (**d**) ProCinch RT (Stryker Sports Medicine, Greenwood Village, Colorado, USA)

The G-Lok (Stryker Sports Medicine, Greenwood Village, Colorado, USA) fixed-loop device was compared against three adjustable-loop devices: UltraButton (Smith & Nephew, Andover, Massachusetts, USA), RigidLoop Adjustable (DePuy Mitek, Raynham, Massachusetts, USA), and ProCinch RT (Stryker Sports Medicine, Greenwood Village, Colorado, USA). All four devices consist of a loop and a locking-button, the loops on the adjustable devices have free ends to adjust the size of the loop (Fig. 1).

**Fig. 2:**
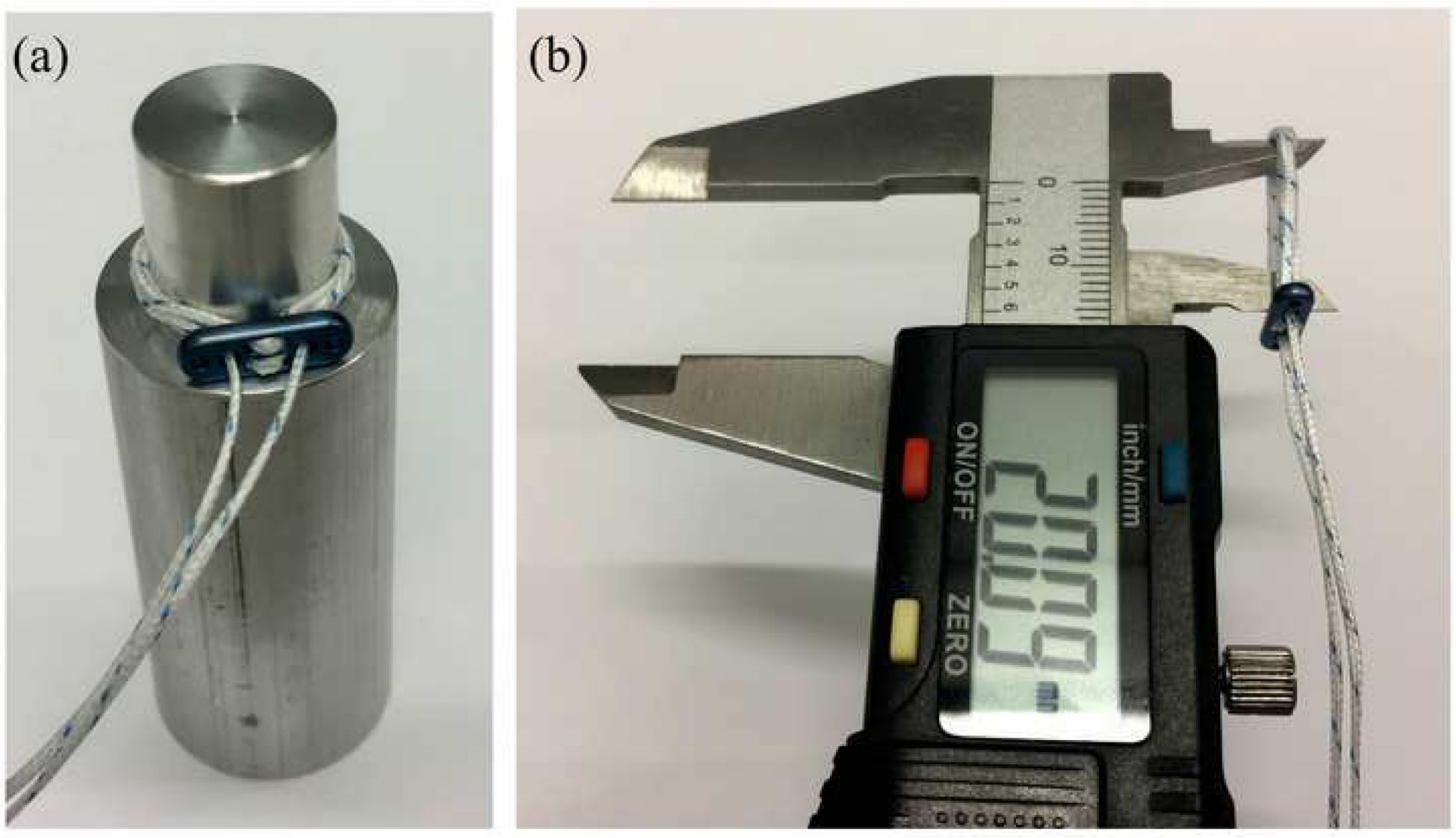
**a** Adjustable-loop devices were tightened to match the size of the fixed-loop device using a custom-built 20 mm diameter cylinder, **b** the size was confirmed using a Vernier calliper

The three adjustable-loop devices were tightened to 20 mm to match the size of the fixed-loop device, using a custom-built 20 mm diameter cylinder, and confirmed using a Vernier calliper (Fig. 2). The devices were adjusted by pulling the free ends with a slow rocking motion, as recommended by the manufacturers [24, 32–34]. A trained technician was required to adjust the UltraButton loop according to the manufacturer’s strict protocol involving additional sideways movements; variations in this technique can affect the performance of this device, and lead to failure during testing.

**Fig. 3:**
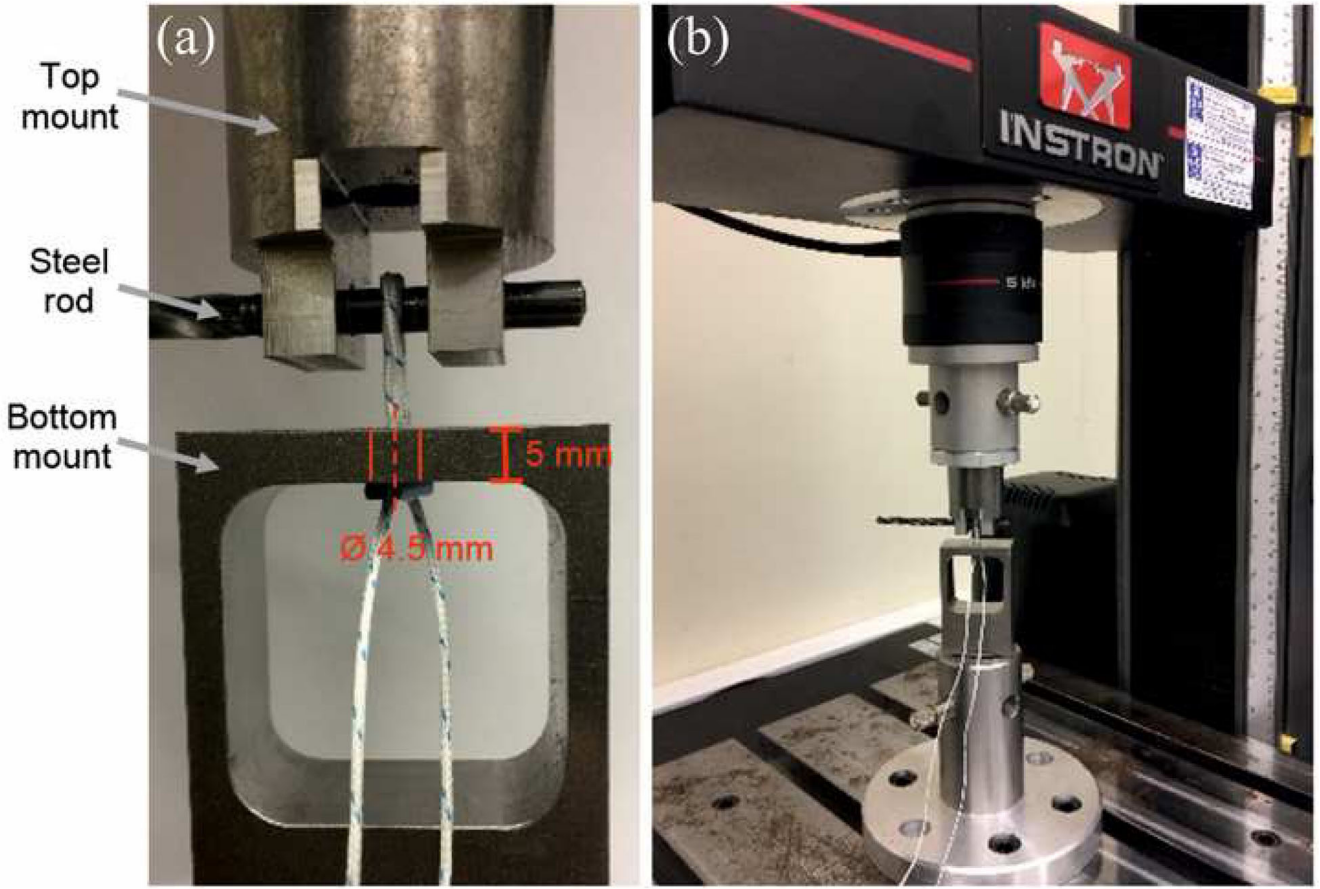
Testing was performed on a simple, custom-built rig, fixed to an Instron machine (Instron, Illinois Tool Works Inc., Norwood, Massachusetts, USA). The rig comprised a bottom-mount attached to the baseplate, a top-mount attached to the crosshead and 5 kN load cell, and a 4.5 mm horizontal steel rod held between two holes in the top-mount

Five devices of each type were tested on a simple, custom-built rig fixed to an Instron machine (Instron, Illinois Tool Works Inc., Norwood, Massachusetts, USA). Similarly to previous studies the rig comprised a bottom-mount attached to the baseplate, a top-mount attached to the crosshead and 5 kN load cell, and a 4.5 mm horizontal steel rod held between two holes in the top-mount (Fig. 3a) [3, 17]. In accordance to the manufacturer’s recommendations for the four devices, the loop of each device was fed upwards through a 5 mm deep and 4.5 mm diameter hole, representing the drilled femoral tunnel, until the button of the loop lay flat against the lower surface [24, 32–34]. The steel rod, representing the graft, was inserted through the loop, avoiding tension in the loop. When the loop was correctly positioned, the crosshead of the machine was moved upwards to remove any slack, until a 1 N load was measured by the load cell (Fig. 3b).

**Fig. 4:**
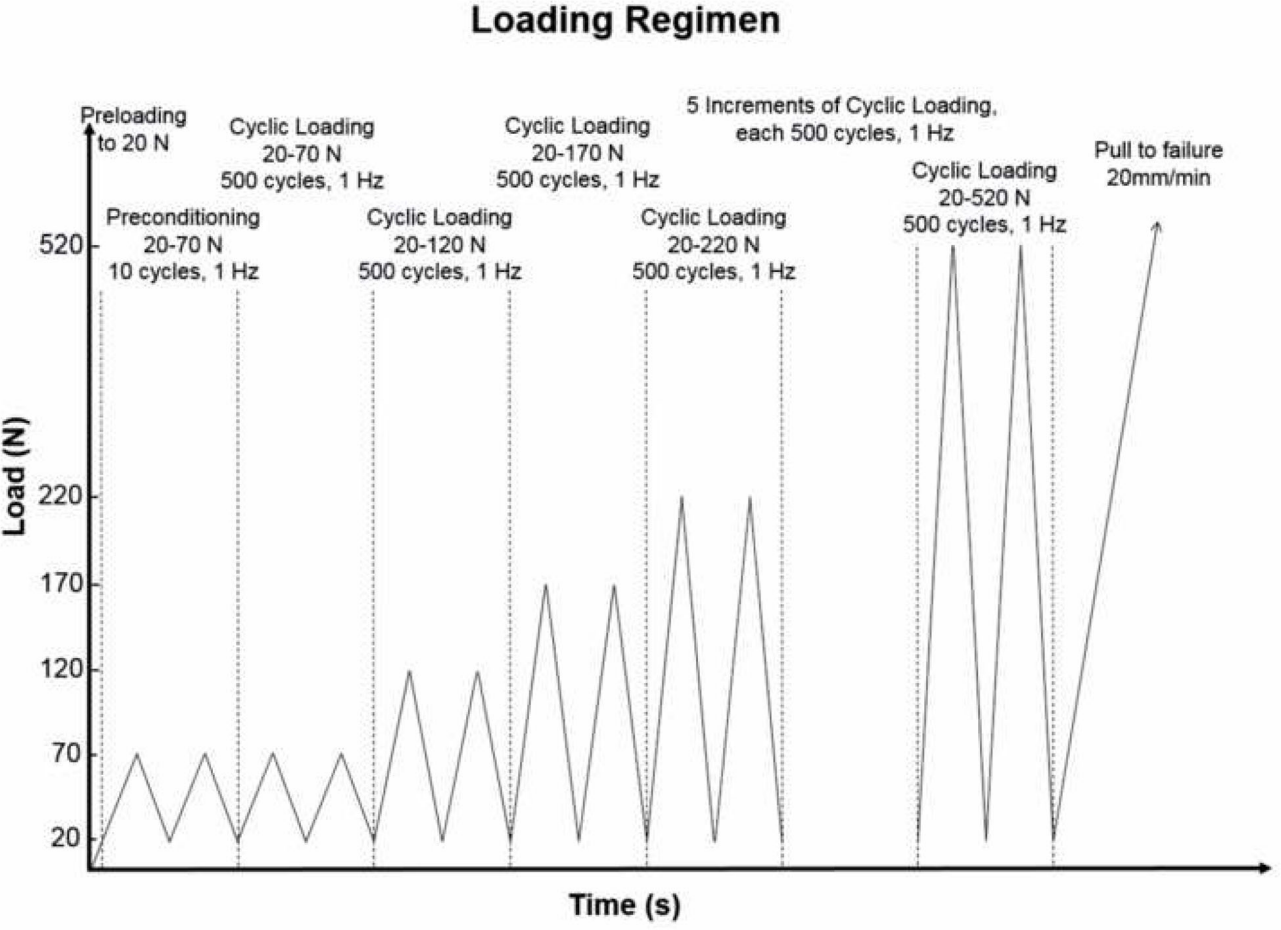
The testing protocol had four stages: preloading, cyclic preconditioning, incremental cyclic loading and pull-to-failure. A 20 N preload was first applied, followed by 10 preconditioning cycles at 1 Hz with loads between 20 and 70 N, incremental loading involved 5000 cycles at 1 Hz with loads between 20 and 520 N, lastly a test to failure was performed at a rate of 20 mm/min

The testing protocol had four stages: preloading, cyclic preconditioning, incremental cyclic loading and pull-to-failure (Fig. 4). A 20 N preload was first applied to simulate intraoperative tensioning, this was followed by 10 preconditioning cycles at 1 Hz with loads between 20 and 70 N, to simulate the surgeon bending the knee before fixation. After preconditioning the loops were re-tensioned using the same technique as during initial tensioning [17]. Upon completion, the elongation of the device was recorded and reset to 0. The incremental loading phase involved 5000 cycles at 1 Hz, with loads between 20 and 520 N, increasing in increments of 50 N, this simulated the forces that occur in the ACL graft during the initial phase of postoperative rehabilitation [28, 30, 35]. A test to failure was then performed at a rate of 20 mm/min.

Load-displacement data was recorded using the Bluehill software (Instron, Illinois Tool Works Inc., Norwood, Massachusetts, USA). Outcome measures were loop elongation after 5000 cycles, loop-elongation at failure, and load at failure.

### Statistical analysis

Using a sample-size calculator for a two-sample t-test (MiniTab Inc., State College PA, USA) at 80% power, it was estimated that testing five samples of each device would allow detection of a 0.3 mm difference in elongation, which represents 10% of the clinical laxity limit, or failure [13, 19].

Descriptive statistics were used to summarise the data. Comparisons between the devices were performed using Kruskal-Wallis tests, for loop elongation after 5000 cycles, elongation at failure and ultimate failure load, in addition, Wilcoxon rank sum tests were used to perform pairwise comparisons between devices, with corrections for multiple testing. Statistical analyses were performed using R version 3.6.1 (R Foundation for Statistical Computing, Vienna, Austria). A *p*-value of 0.05 was used to represent a statistically significant difference.

## Results

The loop elongation after 5000 cycles for the G-Lok fixed-loop device was 1.46 ± 0.25 mm, which was comparable to that of the RigidLoop Adjustable (1.51 ± 0.16 mm, *p* = 1.000) and ProCinch RT (1.60 ± 0.09 mm, *p* = 1.000) (Tables 1 and 2). In comparison, the loop elongation for the UltraButton was 2.66 ± 0.28 mm, which was significantly larger than all other devices (*p* = 0.048).

**Table 1:** Elongation and load data for all cortical suspension devices

	Device type	Elongation during cyclic loading (mm)		Elongation at failure (mm)		Ultimate failure load (N)	
		Mean ± SD	Range	Mean ± SD	Range	Mean ± SD	Range
**G-Lok**	Fixed-loop	1.46 ± 0.25	(1.02 – 1.65)	3.17 ± 0.33	(2.63 – 3.51)	2178 ± 118	(2075 – 2367)
**UltraButton**	Adjustable-loop	2.66 ± 0.28	(2.32 – 3.07)	4.20 ± 0.33	(3.75 – 4.53)	1903 ± 81	(1798 – 1998)
**RigidLoop Adjustable**	Adjustable-loop	1.51 ± 0.16	(1.38 – 1.71)	2.88 ± 0.20	(2.76 – 3.24)	1835 ± 179	(1529 – 1975)
**ProCinch RT**	Adjustable-loop	1.60 ± 0.09	(1.50 – 1.73)	2.78 ± 0.08	(2.70 – 2.92)	1456 ± 137	(1322 – 1668)

*Abbreviations*: *SD* standard deviation

**Table 2:**
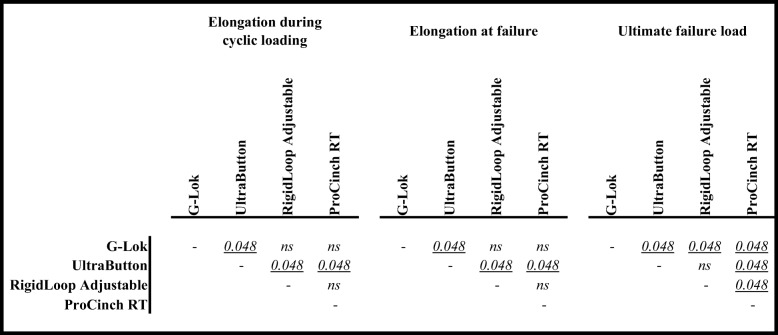
Comparison across cortical suspension devices for elongation and load data

The failure load for all devices ranged between 1455 and 2178 N (Table 1). The G-Lok fixed-loop device was significantly stronger than all adjustable-loop devices (*p* = 0.048), while ProCinch RT was significantly weaker than all other devices (*p* = 0.048) and there was no significant difference between UltraButton and RigidLoop Adjustable (*p* = 0.690). The most common method of device failure, which was seen in all devices, was breakage of the loop at the button level. In addition, the button itself also broke in some samples of G-Lok and UltraButton.

The elongation at failure was largest for the UltraButton adjustable-loop device (4.20 ± 0.33 mm), which was significantly greater than G-Lok (3.17 ± 0.33 mm, *p* = 0.048), RigidLoop Adjustable (2.88 ± 0.20 mm, *p* = 0.048) and ProCinch RT (2.78 ± 0.08 mm, *p* = 0.048) (Tables 1 and 2). There was no significant difference in elongation at failure for the rest of the devices.

**Fig. 5:**
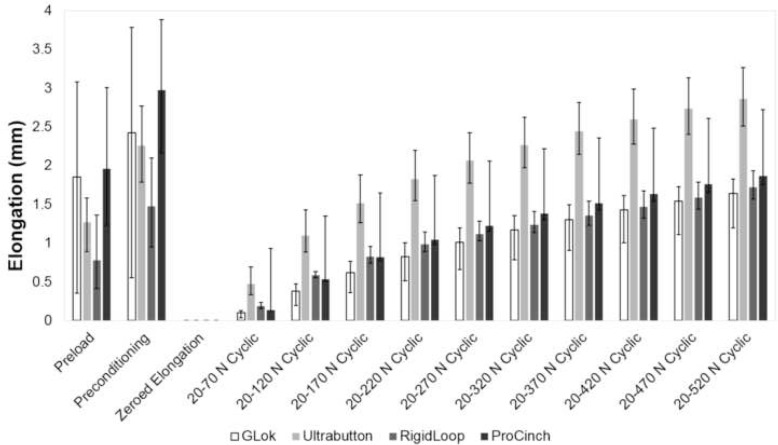
Loop elongation at each loading stage for all devices

**Fig. 6:**
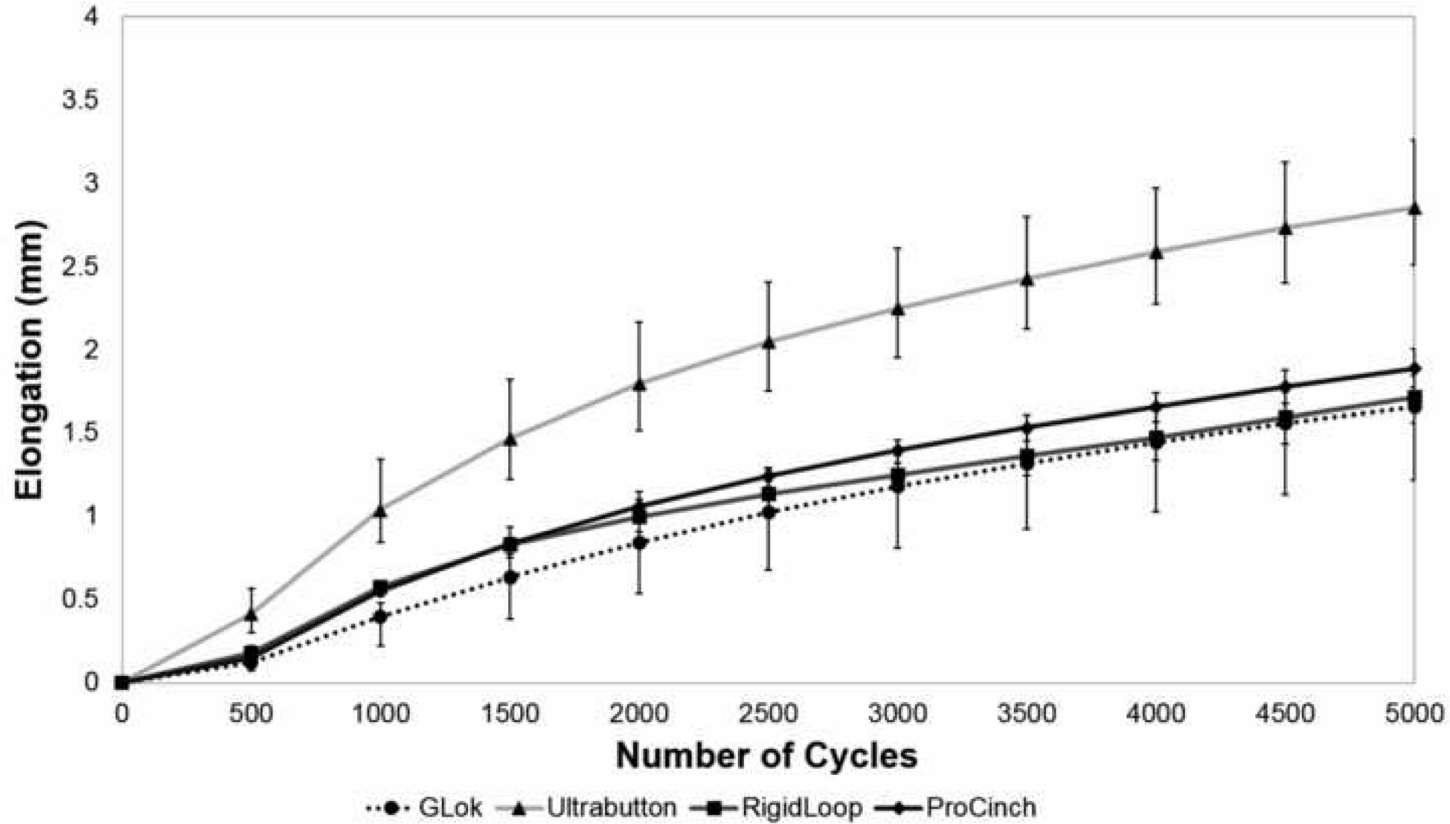
Loop elongation at each cycle of the incremental cyclic loading phase for all devices

The maximum loop elongation occurred for all specimens during the application of the 20 N preload, and the second largest elongation occurred during preconditioning between 20 and 70 N (Fig. 5). The elongation increased incrementally between cycle 1 and 5000 (Fig. 6).

## Discussion

The most important finding of this study is that the fixed-loop G-Lok device and the three adjustable-loop devices all had mean elongations after 5000 cycles that were less than 3 mm. Thus, they all have the necessary biomechanical properties, in terms of reduced loop elongation and high failure load, for initial fixation of soft tissue grafts, when tested under extended cycles and high loads. Our study has been the first to compare the G-Lok cortical suspension fixed-loop device against the Ultrabutton, RigidLoop Adjustable and ProCinch RT adjustable-loop devices over an extended number of cycles (5000) at high loads (500 N), thus more closely replicating the early rehabilitation period in vitro.

Adjustable-loop femoral cortical suspension devices offer several advantages over fixed-loop devices [14, 16], however, there are concerns about their tendency to elongate when subjected to biomechanical loading, which may compromise the effective length of the graft-loop construct [3, 17, 25, 26]. The length of the graft and fixation device construct is critical during the first 8–12 postoperative weeks and early rehabilitation, while the graft heals. Elongation of the device by more than 3 mm could not only lead to clinical instability but may also impair tendon to bone healing [12, 13, 19]. In the current study, all cortical suspension devices elongated by less than 3 mm after 5000 cycles, although a significant difference was observed between the UltraButton and the three other devices. In terms of failure loads, the G-Lok was significantly stronger, while the ProCinch RT was significantly weaker than all other devices. However, failure loads of all devices exceeded the forces measured on ACL grafts during early rehabilitation which have peaks below 500 N [5, 23, 29]. The G-Lok showed the lowest extent of elongation and the highest failure load, thus supporting our hypothesis.

The low elongation of the G-Lok fixed-loop device was consistent with other published studies, most of which used the fixed-loop EndoButton CL (Smith & Nephew, Andover, Massachusetts, USA) as the reference fixed-loop device [2, 3, 17, 25] (Table 3). Rylander et al. have compared the G-Lok against the EndoButton CL and have found no significant differences in elongation between the two devices during cyclic loading to 250 N for 1000 cycles [27].

**Table 3:** Literature table reporting the elongation during cyclic loading and ultimate failure load of both fixed- and adjustable- loop femoral cortical suspension devices

Author	Year	Journal	Model	Fixed-loop devices	Adjustable-loop devices	Company	Sample size	Preconditioning			Cyclic loading			Pull-to-failure (mm/min)	Elongation during cyclic loading (mm)	Ultimate failure load (N)
								No. of cycles	Applied load (N)	Freq. (Hz)	No. of cycles	Applied load (N)	Freq. (Hz)			
									Min. Max.			Min. Max.			Mean ± SD	Mean ± SD
Current study			Device only	G-Lok		Stryker	5	10	20 - 70	1	5000	20 - 520	1	20	1.46 ± 0.25	2178 ± 118
					UltraButton	Smith & Nephew	5								2.66 ± 0.28	1903 ± 81
					RigidLoop	DePuy	5								1.51 ± 0.16	1835 ± 179
					ProCinch	Stryker	5								1.60 ± 0.09	1456 ± 137
Ahmad	2004	AJSM	Porcine construct	Endobutton		Smith & Nephew	8				1000	50 - 250	1	20 cm/min	1.75 ± 0.97	864 ± 164
Barrow	2014	AJSM		Endobutton		Smith & Nephew	6	10	10 - 50	1	4500	10 - 250	1	20	1.34 ± 0.03	1529 ± 26
					ToggleLoc	Biomet	6								5.76 ± 0.35	1652 ± 45
					Tightrope	Arthrex	6								42.45 ± 7.01	809 ± 53
Chang	2018	Arthroscopy	Device only	Endobutton		Smith & Nephew	6	10	10 - 75	0.1	4500	100 - 400	0.5	50	0.74	1410
					Tightrope	Arthrex	6								1.91	925
			Porcine construct	Endobutton		Smith & Nephew	6	10	10 - 75	0.1	4500	100 - 400	0.5	50	14.88	843
					Tightrope	Arthrex	6								15.65	888
Conner	2010	Arthroscopy	Porcine construct	Endobutton		Smith & Nephew	8				2000	50 - 450	1	30 mm/s	3.55 ± 0.57	1191 ± 150
					ToggleLoc	Biomet	8								Data not given.	
					EZLoc	Biomet	8								5.46 ± 0.95	913 ± 82
Johnson	2015	AJSM		Endobutton		Smith & Nephew	8	10	10 - 75	0.1	1000	100 - 400	0.5	50	1.05 ± 0.05	1530 ± 180
				XO Button		ConMed Linvatec	8								1.65 ± 0.43	2218 ± 114
				Rigidloop		DePuy	8								1.09 ± 0.16	1976 ± 229
					Tightrope	Arthrex	8								2.2 ± 0.62	784 ± 45
					ToggleLoc	Biomet	8								3.69 ± 2.39	1995 ± 217
Noonan	2016	Arthroscopy	Device only	Endobutton		Smith & Nephew	5	25	50 - 250		4500	50 - 250			0.52 ± 0.08	1384 ± 71
					Tightrope	Arthrex	5								0.96 ± 0.07	886 ± 39
			Porcine construct	Endobutton		Smith & Nephew	5	25	50 - 250		1000	50 - 250			3 ± 0.30	866 ± 102
					Tightrope	Arthrex	5								2.7 ± 0.50	786 ± 166
Petre	2013	AJSM	Device only	Endobutton		Smith & Nephew	5	10	10 - 50	0.1	1000	50 - 250	0.5	50	0.11 ± 0.03	1456 ± 130
				XO Button		ConMed Linvatec	5								0.35 ± 0.06	2230 ± 252
					Tightrope	Arthrex	5								0.3 ± 0.04	841 ± 55
					ToggleLoc	Biomet	5								0.82 ± 0.18	1561 ± 112
			Porcine construct	Endobutton		Smith & Nephew	10	10	10 - 50	0.1	1000	50 - 250	0.5	50	1.88 ± 0.25	1456 ± 101
				XO Button		ConMed Linvatec	10								1.82 ± 0.23	1748 ± 140
					Tightrope	Arthrex	10								2.74 ± 0.39	859 ± 43
					ToggleLoc	Biomet	10								3.34 ± 1.28	1334 ± 81
Rylander	2014	Clinical Biomechanics	Porcine construct	G-Lok		Stryker	10				1000	50 - 250	1	20		614 ± 176
				Endobutton		Smith & Nephew	10									717 ± 128
					ToggleLoc	Biomet	10									560 ± 101
					RetroButton	Arthrex	10									526 ± 160

Chang et al. [7] are the only previous authors to use a similar testing protocol to the current study, with both an extended number of cycles (4500) and high loading (100–400 N). Chang et al. [7] compared the biomechanical properties of two different cortical suspension devices, the fixed-loop EndoButton CL and the adjustable-loop TightRope RT (Arthrex, Naples, FL) and their results were in good agreement with the current study, the fixed-loop device had a higher tensile strength and elongated less than the adjustable-loop device (*p* = 0.001), although both devices elongated less than 3 mm.

A number of other studies have used either an extended number of cycles [3, 25] or a high loading [2, 9], and similarly to the current study they have all reported significantly smaller elongations for fixed-loop cortical suspension devices in comparison to adjustable-loop devices, both when using a device-only model and a device-bone-soft tissue construct model [3, 9, 17, 25]. A few of the devices tested by these authors exceeded an elongation of 3 mm, thus resulting in clinical failure (Table 3). In agreement with our study, Chang et al. [7], Conner et al. [9] and Noonan et al. [25] also found that fixed-loop devices had significantly greater ultimate failure loads than adjustable-loop devices.

Similarly to the studies by Barrow et al. [3] and Noonan et al. [25], our study also found that the greatest amount of loop elongation occurred at low loads, during preloading (20 N) and preconditioning (20–70 N). Loop elongation at low loads has clinical implications, because the ACL is subject to low loads (0–20 N) in some ACL reconstruction rehabilitation exercises, such as dynamic squat to stand at 25 degrees, barbell squats and leg press [10, 29, 35]. Nonetheless, it is possible that in early rehabilitation the forces on the ACL are not sufficient to cause elongation, or that the cycling of graft and fixing at the tibial side with graft under tension mitigates the effects of elongation in the initial cycles [29, 37].

Although in vitro studies, including our own, have shown a greater elongation of adjustable-loop devices in comparison to fixed-loop devices, there is no clinical study showing significant differences in laxity between the two types of devices [4, 6, 8, 11, 21, 37]. The main limitations of this study are that testing was done in vitro, and we were unable to simulate in vivo conditions such as graft healing, the role of supporting structures, bone density and line-of-pull [3, 13, 17, 19, 31]. However, by testing the devices in vitro confounding variables such as bone quality were removed. In addition, only 5 samples of each device were tested, although this was enough to produce a statistical power above 80%. It is important to note that the protocol for implantation of the UltraButton was more complex compared to the other devices, which could affect clinical outcomes if the surgeon is not properly trained. The main strength of our study was the controlled testing of loops under high loads over an extended number of loading cycles.

## Conclusions

The purpose of this study was to compare the biomechanical properties of the G-Lok fixed-loop device against three adjustable-loop devices during testing over 5000 cycles under high loading. Our study has shown that all devices have the necessary biomechanical properties for initial fixation of soft tissue grafts in the femoral tunnel for ACL reconstruction. Nonetheless, the fixed-loop device performed best in terms of least elongation and highest load at failure.

## Abbreviation

ACL: Anterior cruciate ligament
